# Relevance of immune cell and tumor microenvironment imaging in the new era of immunotherapy

**DOI:** 10.1186/s13046-020-01586-y

**Published:** 2020-05-18

**Authors:** Filippo Galli, Jesus Vera Aguilera, Belinda Palermo, Svetomir N. Markovic, Paola Nisticò, Alberto Signore

**Affiliations:** 1 Nuclear Medicine Unit, Department of Medical-Surgical Sciences and of Translational Medicine, “Sapienza” University of Rome, S. Andrea University Hospital, Roma, Italy; 2 Department of oncology and Department of Immunology, Mayo Clinic, (MN), Rochester, USA; 3 Tumor Immunology and Immunotherapy Unit, Department of Research, Advanced Diagnostics and Technological Innovation, IRCCS Regina Elena National Cancer Institute, Rome, Italy

**Keywords:** molecular imaging, tumor microenvironment, onco-immunology, immunotherapy, lymphocytes

## Abstract

Tumor-infiltrating immune cells play a key role against cancer. However, malignant cells are able to evade the immune response and establish a very complex balance in which different immune subtypes may drive tumor progression, metastatization and resistance to therapy. New immunotherapeutic approaches aim at restoring the natural balance and increase immune response against cancer by different mechanisms. The complexity of these interactions and the heterogeneity of immune cell subpopulations are a real challenge when trying to develop new immunotherapeutics and evaluate or predict their efficacy in vivo. To this purpose, molecular imaging can offer non-invasive diagnostic tools like radiopharmaceuticals, contrast agents or fluorescent dyes. These agents can be useful for preclinical and clinical purposes and can overcome [^18^F]FDG limitations in discriminating between true-progression and pseudo-progression. This review provides a comprehensive overview of immune cells involved in microenvironment, available immunotherapies and imaging agents to highlight the importance of new therapeutic biomarkers and their in vivo evaluation to improve the management of cancer patients.

## Background

Immunotherapy is the most appealing anti-cancer approach of the modern era and researchers are continuously exploring new ways to reprogram immune cells of the host against cancer [1]. Despite the initial hype, due to promising results, playing with the immune system raised important issues in many treated patients together with controversial results. Indeed, the removal of the intrinsic immune suppression can trigger a cascade of events with serious adverse effects [2].

Moreover, because of the complex and dynamic nature of the interactions between cancer and immune cells, a high inter- and intra-patient heterogeneity is observed, sometimes leading to failure of the treatment [3]. That is why there is an urgent need of diagnostic tools to help physician in predicting and evaluating treatment response at very early stages. This will help to accurately select patients for specific therapies and to promptly suspend or change the therapeutic approach if needed. Indeed, the possibility to characterize in vivo each tumor lesion opens the door to true personalized-medicine that we might even define as “lesion based-medicine” [4].

In this scenario, molecular medicine imaging offers plenty of tools to specifically follow immune cell subtypes in a non-invasive manner [5]. This is not only thanks to availability of many radiopharmaceutical and probes to target specific cell subtypes, but also to high sensitivity technologies that can allow us to detect even limited numbers of cancer infiltrating cells. In this review we will give an overview of tumor microenvironment, new therapies and the added value of molecular imaging towards a personalized medicine approach.

### The tumor microenvironment

**Fig. 1:**
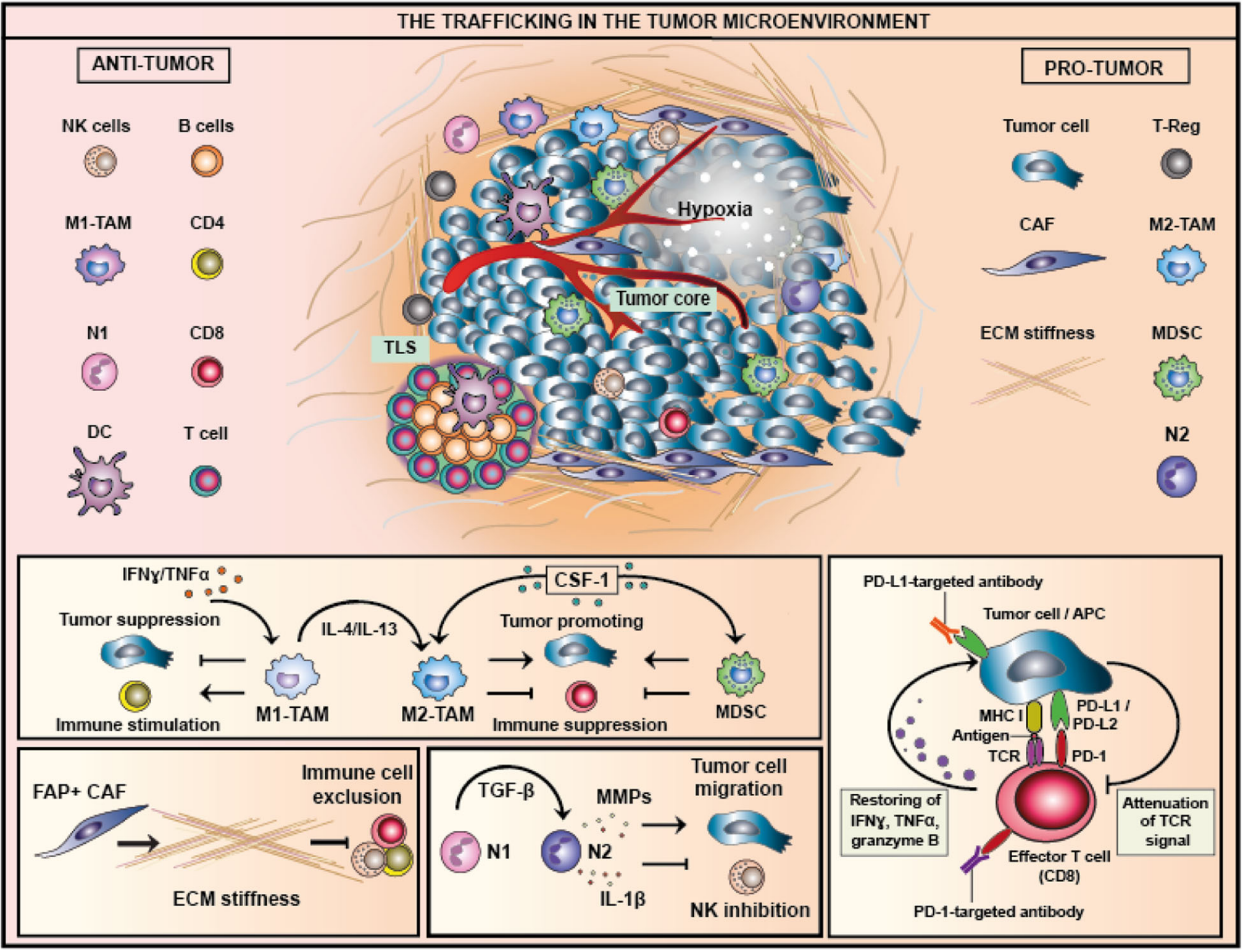
The trafficking in the tumor microenvironment

Cancers are not a mass of transformed cells but rather a new organ composed of various non-malignant cells comprising a large portion of the tumor mass, which have become wayward and lost the ability to maintain a dialogue enabling homeostasis of the tissue architecture [6]. These cells include fibroblasts, adipocytes, pericytes, vascular endothelial cells, and, as main players, immune cells [7]. Tumor and stromal cells co-evolve, similarly to what occurs in organogenesis during development, and the interaction among the different components leads to a continuous phenotypic and functional plasticity. Dynamic reciprocal communication between cells and microenvironment is conducted via junctions and receptors plus a plethora of signals produced by the multiple cell types encased in a three-dimensional extracellular matrix (ECM). This includes glycoproteins, proteoglycans, cytokines and growth factors, together with ECM-remodeling enzymes, providing both structural support and appropriate information [8]. The disruption of tissue homeostasis creates dynamic changes in the cellular metabolism and function of both stromal and immune cells [9]. This highly trafficked network constitutes the tumor microenvironment (TME) (Fig. 1), and cancer research has to make a major effort to draw up a multidimensional map that will elucidate the highways and byways of the cancer battlefield.

### Cells of the tumor microenvironment

#### Immune cells - T lymphocytes

T lymphocytes are the most potent mediators of adaptive anti-tumor immune response. The cytotoxic CD8+ T cell population, supported by CD4+ T helper (Th1) cells through the production of IL2 and IFNγ, generates the final effector mechanism leading to tumor elimination and are associated with a good prognosis [10, 11]. Whereas, the CD4+ T cell subsets Th2 and Th17, producing IL4, IL5, IL13 or IL17A, IL17F, IL21 and IL22 respectively, are generally associated with tissue inflammation and a pro-tumorigenic effect. The CD8-mediated immune response is modulated by an immunosuppressive class of CD4+ T cells known as T regulatory (Treg), which expresses the CD25 and FOXP3 molecules, governing peripheral immune tolerance [12]. In the TME, high amount of Tregs is often present and their main role is to suppress the anti-tumor response. However, due to the Treg subpopulation diversity along with different functional pathways, their role in cancer development and progression is ambiguous and still not fully understood [13, 14].

The successful control of tumor progression, mediated by T lymphocytes, firstly requires that they infiltrate the tumors. In fact, the immune contexture and the T cell abundance, functional activity and spatial distribution in the TME are crucial prognostic and predictive factors [15], as recently proposed for the immune checkpoint blockades (ICB) [16, 17].

Compartmentation of the immune response into three major phenotypes - inflamed, immune-excluded and immune-desert phenotypes - has been proposed as the major predictor of response to different cancer treatments in the new era of immune inhibitory receptor blockades [17, 18]. The inflamed phenotype comprises the concurrent presence of both CD8+ and CD4+ T cells with inhibitory cells (i.e., macrophages, fibroblasts, Treg, suppressor myeloid cells and B cells) in the tumor parenchyma. These cells affect T cell functionality up-regulating several inhibitory receptors and leading to T cell dysfunction and exhaustion [19]. The immune-excluded phenotype has been associated to mesenchymal traits, which have been proposed as putative biomarkers of response to ICB [20]. This phenotype is characterized by a huge number of immune cells in the stroma surrounding tumor nests, as dictated by physical barriers (i.e., stiffened tissue with high matrix fiber mass and dense collagen network) [21] or the low expression of specific chemokines involved in T cell recruitment [22, 23].

The above-mentioned suppressive cells, accompanied by the hindrance of lymphocyte infiltration and trafficking, may be modulated in multiple ways. Indeed, soluble molecules, such as vascular endothelial growth factor (VEGF) and the consequent abnormal neovasculature [24] as well as down-modulation by tumor cells of adhesion and chemotactic signals on the tumor endothelium, may participate in an immune-suppressive TME [25]. T cell exclusion may also be mediated by cancer-associated fibroblasts, which produce the C-X-C motif chemokine 12 (CXCL12). This chemokine inhibition in a mouse model of pancreatic ductal adenocarcinoma (PDAC) has been shown to revert the immune exclusion and synergize with the anti-PD1 therapy [26].

The lack of an endogenous anti-tumor response in TME described as the third immune-desert phenotype, may be due to insufficient T cell priming, immunological ignorance or induction of tolerance. This immune contexture is characterized by the presence of Treg, MDSC and macrophages, which wire a circuit inhibiting dendritic cell (DC) maturation and hamper T cell expansion and activation [27].

#### B lymphocytes

Recent findings have assessed a role for B cells in the anti-tumor immune response [28] B cell infiltration into the TME occurs as occasionally localized at the invasive margin of tumors, but more often localized in draining lymph nodes and tertiary lymphoid structures (TLS), and may be associated to both positive and negative effects in tumor immunity. The anti-tumor role of B cells has been reported in murine models, indicating that B cells increase T cell functionality [29]. In different human tumors, such as ovarian, non-small cell lung cancer (NSCLC), gastric and cervical cancer, the presence of tumor-infiltrating CD20+ B cells is associated with good prognosis [30–33]. Despite this protective role, B cells may negatively regulate anti-tumor immunity, as reported in a murine model of squamous carcinogenesis [34]. Similarly, Ammirante et al. showed that B cells, recruited by the chemokine CXCL13, promote the progression of castrate-resistant prostate cancer by producing lymphotoxin [35]. Furthermore, the immunogenic effect of chemotherapy in mouse and human prostate tumors, requires the removal of an immunosuppressive B cell subtype, plasmocytes that express IgA, interleukin (IL)-10 and programmed death ligand 1 (PD-L1) - the appearance of which depends on TGFβ receptor signalling - that induce CD8+ T cell exhaustion and suppress anti-tumor CTL responses [36].

#### Natural killer cells

Tumor stroma may be infiltrated by innate cytotoxic lymphocytes, the natural killer (NK) cells. NK cells not only recognize and kill cancer cells through the release of cytolytic granules, but also greatly impact the adaptive anti-tumor immune response by producing chemokines and cytokines. NK cells are highly heterogeneous, and the availability of different combination markers has allowed researchers to identify distinct subpopulations with definite functionality [37]. Otherwise, in common with tumor-associated immune cells, NK cells can also negatively influence anti-cancer responses by modulating DC and T cells. Recently, Glasner et al. reported a new anti-tumor role of NK cells by modulating the immune response. The authors demonstrated that the activation of the NK natural cytotoxic receptor 1 (mouse) and NKp46 (human) induces IFNγ production which, in turn, modulates fibronectin 1 expression on tumor cells, preventing metastatic spread [38]. However, different tumor-related soluble factors (i.e. IL-10, IDO, PGE2, TGF-β1) produced by different tumor-infiltrating immune cells (i.e. M2-macrophages, MDSC, DC, Treg), may negatively affect NK cell activity [39].

#### Dendritic cells

DCs are antigen-presenting cells (APC) able to capture antigens in the form of peptide-major histocompatibility (MHC) molecule complexes and present them to the T cells [40]. They are a ubiquitous population of myeloid cells, heterogeneous in terms of morphology, ontogeny and immunological features [41]. The different DC subsets are related to specific immunological functions: a) DC processing and presenting antigens; b) epidermal Langerhans cells specializing in priming CD8+ T cell immunity and interstitial/dermal (CD14+) DCs endorsing humoral immunity; c) plasmacytoid (pDCs) secreting high amount of type I IFN. DCs exist in immature state (iDCs) in the absence of maturation signals, eliciting immunological tolerance and/or suppression. Several cues, such as microbe-associated molecular patterns or endogenous damage-associated molecular patterns, can lead iDC to a mature state [42].

#### Tertiary lymphoid structures

TLS are lymphoid aggregates induced postnatally in non-lymphoid tissues that resemble the organization of lymph nodes, characterized by clusters of mature DCs and T cells juxtaposing B-cell follicles and high endothelial venules without encapsulation. Similar to lymph nodes, they are assumed to provide the main lymphocytic functional environments for both cellular and humoral immunity [43]. The TLS architecture is coordinated by homeostatic chemokines, i.e. CCL19, CCL21, CXCL13 and CXCL12, the same found in the secondary lymphoid organ. The presence of peri- and/or intra-tumoral TLS has been correlated with a good prognosis and prolonged patient's survival in 12 different types of cancer. Further studies are needed to elucidate the immune mechanisms that are activated within these structures and the driver mechanisms of their development within the tumor. From the clinical point of view, it is urgent to understand whether the presence and localization of TLS in pre-treatment or longitudinal tumor tissue samples during and post-treatment, may be validated as prognostic/predictive of responses to checkpoint blockade, with far reaching clinical implications, as recently reported [44, 45].

#### Macrophages

Most of the immune cell populations within the tumor stroma are made up of tumor-associated macrophages (TAMs), major players in orchestrating cancer-related inflammation. Pre-clinical and clinical evidences demonstrated that an abundance of TAMs in the TME is associated with a poor prognosis [46]. The bi-directional communication between macrophages and TME affects their phenotype, and is strictly dependent on the disease stage and the involved tissue. Indeed, pro-inflammatory macrophages, which play a key role against pathogens are driven by cytokines, such as IFNγ, TNFα and microbial products, and are referred to as the M1 subtype. This subtype in turn favours a Th1 response. On the other hand, IL-4 or IL-13 determine the M2 subtype polarization, related to tumor-promotion and contributes to an immune-suppressive TME, hampering T cell functionality [47]. TAMs have shown to negatively affect T cell responses in hepatocellular [48] and ovarian cancer [49], through PD-L1 and B7-H4, respectively. Besides, TAMs can also express PD-L2 along with B7-H4 and VISTA immune checkpoint inhibitory molecules. Overall, the activity of TAMs in cancer is usually pro-tumorigenic, closely related to the colony-stimulating factor (CSF)-1 secretion by cancer cells that recruit TAMs, which in turn, by releasing EGF, edit cancer cells and favour cell migration, extravasation and metastases [50]. Several pharmacological agents targeting macrophages in tumor have been successfully tested in experimental tumor indicating the rationale to move into clinical trial [51].

#### Neutrophils

Neutrophils constitute 50-70% of all circulating leukocytes and representing the traditional front line of defense against infection. Inside the TME, a number of key molecular mechanisms can promote neutrophil polarization in two opposite subpopulations of anti-tumorigenic (N1) and pro-tumorigenic (N2) tumor-associated neutrophils (TANs) [52]. In particular, TGF-β secreted by cancer associated fibroblasts (CAFs) is responsible for both the recruitment and activation of N2 [53] and the suppression of N1 neutrophils [54]. It has been suggested that the degree of tumor development is the primary determinant of the resulting TAN phenotype [55]. N2 TANs through the secretion of MMPs and interleukin (IL)-1β activates endothelial cells and inhibits NK cells, promoting tumor cell plasticity [56] and cancer migration [57]. Disseminating cancer cells interact with neutrophils in the metastatic sites and it is crucial to understand the neutrophils contribution to the metastatic processes, keeping in mind that many cancer patients who are undergoing chemotherapy are also treated with neutrophil-stimulating factors [58]. Furthermore, in melanoma patients, high levels of circulating neutrophils and neutrophil-to-lymphocyte ratio have been associated with resistance to anti-CTLA-4, indicating the main role that these cells may exert in inhibiting immune response [59].

#### Myeloid-derived suppressor cells

Myeloid-derived suppressor cells MDSCs are classed as one of the major sub-populations of inhibitory immune cells that are frequently found in several mouse and human cancers and show a plastic phenotype [60, 61] rendering their tracking difficult. Monocytic-MDSCs induce processes related to cancer invasion, such as the epithelial mesenchymal transition (EMT), a dynamic process regulated by microenvironmental stimuli [62] promoting tumor invasiveness by impairing anti-tumor innate and adaptive responses [63]. On the contrary, granulocytic-MDSCs suppress EMT [64]. Furthermore, the presence of MDSC in TME has been linked to ECM modification, as shown in a murine model of breast cancer, highly expressing the secreted protein acidic and cysteine rich [65]. Of clinical relevance, these cells are clearly involved in resistance to ICB therapy in patients [66]. MDSC tumor-infiltration is mediated by CSF-1 and the combination of CSF-1/CSF-1R signalling inhibition with anti-CTLA-4 has been recently proposed [67].

### Cancer associated fibroblasts

Among the non-neoplastic cells in the TME, CAFs are the most prominent stromal component and key players in cancer progression [68]. CAFs secrete growth factors with the TGF-β as the major player favouring EMT through the biomechanical and biochemical remodelling of the ECM. In the context of tumor-stroma coevolution, CAFs are linked to cancer progression, giving mesenchymal traits to tumor cells and contribute to therapeutic outcome [69]. Among the soluble factors produced by CAFs, the IL-6 cytokine mediates a dynamic crosstalk between tumor cells and CAFs, driving mesenchymal tumor phenotype and chemo-resistance [70]. The major contribution of fibroblast composition is their ability to secrete ECM components and its remodelling enzymes [69, 71]. Fibroblast activation protein is expressed in a CAF subtype associated with ECM remodelling and tumor-promoting inflammation [72, 73]. Depletion of these cells determines INFγ production, reverting immunosuppression [74].

### The immunosuppressive TME: ECM, hypoxia and metabolism

The complex mixture of immune cells, non-cancerous cells and cancer cells are embedded in the extracellular matrix. The dysregulation of ECM composition, structure, stiffness and quantity, by regulating mechanical and biochemical cues in the TME, is crucial in cancer progression, invasion and immunosuppression [75]. A recent elegant work has reported an ECM-associated molecular signature predictive of the extent of the disease in ovarian cancer [76].

ECM deposition and remodelling are strictly linked to a reduction of the oxygen level, known as hypoxia. Rapid growth and poor vasculature development frequently lead to hypoxic microenvironments within the tumor. The association between hypoxia and ECM remodelling is mediated by hypoxia inducible factor-1 and 2, which regulate the expression of enzymes related to bio-synthesis fibres in collagen degradation [77]. To survive in hypoxic conditions, cancer cells adopt strategies of metabolic shift from oxidative phosphorylation to glycolysis [78]. Glycolysis within tumor cells has been reported to compete with glucose availability to T cells, associated with an inhibition of effector function [79]. These data pave the way for new studies aimed at measuring the effect of ICB therapy on available intra-tumoral nutrients for immune cell metabolism in treated patients and their clinical response.

The overview of this amazing complexity surely justifies a great multidisciplinary effort in cancer research and new methodologies to track the immune cells in the highly trafficked highways and byways of the cancer road map.

### Cancer immunotherapy

#### Drugs stimulating the host immune response

The ability of the host immune system to identify and eradicate malignant cells with minimal systemic toxicity remains the holy grail of cancer immunotherapy [80]. The first immunotherapy for the treatment of malignant tumors began in 1891 by William B. Coley (Coley’s toxin). Dr. Coley injected live bacteria (streptococcal organisms) directly into tumors, muscle tissue or intravenously, in patients with soft tissue sarcomas “in order to cause erysipelas and stimulate the immune system” to attack the cancer [81]. Severe toxicity and lack of reproducible results ultimately, in the face of emerging clinical use of chemotherapy and radiation therapy ultimately lead to discontinuation of its use 40 years later [82]. Nonetheless, Coley’s early observations remain as the foundation of cancer immunotherapy to this day, suggesting that activation of immunity can indeed result in tumor rejection. The first of the modern applications of Coley’s principle came about in the 1970s when Morales et al. established the effectiveness of the bacterium Bacillus Calmette-Guérin (BCG) in the treatment of superficial bladder cancer [83]. The underpinnings for this clinical trial include a 1959 study by Old et al. showing the anti-tumor effects of BCG in a mouse model [84]. Besides his work on BCG, Old also performed extensive research and was involved in the description of tumor necrosis factor in 1975 [85]; however the idea that the immune system could play an important role in the treatment of many cancers still remained a concept solidly external to the purview of mainstream oncology [86].

The discovery and characterization of dendritic cells by Ralph Steinman in 1973, the description of MHC restriction in 1974 by Zinkernagel and Doherty’s, the documentation of NK cell activity in 1975 by Eva Klein’s, the investigation in large-scale of cytokines in breast cancer, renal cell cancer (RCC), glioblastoma, lymphoma, and melanoma in the 1980s, initiated the modern immune-based cancer treatments in clinical medicine [86, 87].

#### Monoclonal antibodies

During the past 20 years, mAbs have been a major component of treatment for many cancers, including breast, lymphoma, and colo-rectal cancer malignancies [88]. The prospect of using human mAbs for the prevention or treatment of human diseases was evident early on and was the driving force behind intense effort put into the development of human hybridoma methods [89].

The challenge of identifying antigen-specific cells and expanding them to numbers that enabled researchers to overcome the barrier of low fusion efficiency would, however, require several more decades of investigation. The principal advantage of the use of human hybridoma technology for mAb generation is that this approach preserves the authentic sequence and pairing of antibody DNA from a natural B cell for the expression of a naturally occurring full-length human mAb [90]. Therapeutic mAbs are typically of the IgG class and are composed of a fragment antibody-binding and a fragment constant component. A mAb can be “naked,” meaning it is not combined with any other drug, or conjugated. Conjugated mAbs are joined with chemotherapy drugs, radioactive particles, or toxins so that they can act as a tool to lead these agents into cancer cells [91, 92]. The Food and Drug Administration (FDA) has approved many therapeutic mAbs to treat different types of cancer. In 1997, rituximab (Rituxan, Genentech) became the first mAb approved for clinical use, indicated in patients with selected B-cell malignancies. Numerous other mAbs have been approved since then, among them trastuzumab (Herceptin, Genentech), alemtuzumab (Campath, Genzyme), ibritumomab tiuxetan (Zevalin, Spectrum Pharmaceuticals), cetuximab (Erbitux, Lilly), bevacizumab (Avastin, Genentech), panitumumab (Vectibix, Amgen), ofatumumab (Arzerra, Novartis), ipilimumab (Yervoy, Bristol-Myers Squibb), brentuximab vedotin (Adcetris, Seattle Genetics), nivolumab (Opdivo, Bristol-Myers Squibb), and pembrolizumab (Keytruda, Merck Sharp & Dohme Corp.). Others are under regulatory reviewing at the FDA or are in phase III clinical trials. In 2017, pembrolizumab, an anti-PD-L1 antibody, received approval for any solid tumor with microsatellite instability or mismatch repair deficiency (dMMR) [92]. This is discussed in more details in the next paragraph.

#### Immune checkpoint inhibitors

Immune checkpoint inhibitors constitute an important breakthrough positively influencing treatment outcomes in cancer patients [93]. Treatment with checkpoint inhibitors involve antibodies generated against the cytotoxic T lymphocyte associated protein 4 (CTLA-4), the programmed death receptor 1 (PD-1) or its ligand; thus, immune checkpoint inhibitors modulate the interaction between tumor cells and cytotoxic T lymphocytes in the TME [94]. Targeting with CTLA-4, PD-1 or PD-L1 antibodies reverses the exhaustion of cytotoxic T lymphocytes thus leading to the elimination of tumor cells via the re-induction of the “natural” function of the T cell population. Interestingly, some of the clinical results when using anti PD-1 and anti PD-L1 antibodies may be also due to additional effects on T cells including their targeting of B7.1 [95].

Brunet and colleagues in 1987 described for the first time CTLA-4, also known as CD152, a co-inhibitory molecule that functions to regulate T cell activation and its effect in melanoma were described by Jim Allison’s group in 1995; fourteen years later the FDA approved the revolutionary checkpoint inhibitor ipilimumab a mAb for the treatment of stage IV melanoma.

More recently, the PD-L1 interaction was described as a major pathway used by tumors to suppress immune control [96]. PD-1 receptor (encoded by the gene Pdcd1) is an Ig superfamily member related to CD28 and CTLA-4. It is expressed on the cell surface of activated T cells under normal conditions, by binding to its ligand (PD-L1 and PD-L2), PD-1 down-regulates T cell activation and therefore dampens unwarranted and excessive immune responses, including autoimmunity [97]. The interaction between PD-L1 expressed on tumor and stromal cells and PD-1 on T cells can trigger inhibitory signalling pathways that reduce effector cell functions and T cell-killing capacity [96]. Blocking the PD-1/PD-L1 with mAbs has been shown to potentiate tumor-specific CD8+ T cell infiltration and effector T cell activation that promote tumor rejection [98, 99].

Anti PD-1 or anti PD-L1 antibodies are currently registered by the FDA for metastatic malignant melanoma, non-small cell lung cancer (NSCLC), renal cell cancer, head and neck cancer, urothelial carcinoma and Hodgkin’s lymphoma in various stages of the respective disease and in the context of varying treatment histories [83]. Many other malignancies (e. g. hepatocellular carcinoma, ovarian cancer, mesothelioma, gastric cancer, B cell non-Hodgkin lymphoma) are currently under clinical investigation to determine a possible efficacy of checkpoint inhibition [94, 100].

Anti-CTLA-4 antibodies (ipilimumab and tremelimumab), anti-PD-1 antibodies (nivolumab and pembrolizumab), and anti-PD-L1 antibodies (atezolizumab, avelumab and durvalumab) have produced remarkable results regarding tumor control in many malignancies; however, response is often followed by relapse and disease progression.

In this context, potential antitumor targets are regulatory T cells (Treg cells). It was proposed that they impair activation, survival and expansion of antitumor T cells through the production of immunosuppressive cytokines, such as transforming growth factor-β (TGFβ) and interleukin-10 (IL-10), and the CTLA4 [101]. Depletion of Treg cells or disruption of their differentiation may restore anti-tumour T cell responses and immunosurveillance against cancer cells in mice [101]. Although increased intra-tumoural expression of chemokines such as CC-chemokine ligand 17 (CCL17), CCL22 and CCL28 facilitates the recruitment of Tregs, it is still unclear how the TME supports excessive Tregs suppressive activity or whether their differentiation from naive or effector CD4+ T cells takes place in the TME [101, 102].

### Drugs promoting immune cell recruitment into the tumor

Inflammatory infiltrates in tumors are considered to be a host attempt at the detection of emerging tumor cells and their elimination, for this reason researchers are trying to identify new drugs to increase this immunological infiltrate [103].

#### Oncolytic viruses

For this purpose, viruses have been used based on the observation that some of them could infect and kill leukemic peripheral blood cells in vitro [104]; while most oncolytic viruses are given by direct injection into established tumors, several viruses can be delivered by the intravenous route avoiding the need for tumor localization and/or complex interventional administration strategies [105]. To date, the virus that has gained the most attention is an attenuated herpes simplex virus, type 1 (HSV-1) engineered to express human granulocyte-macrophage colony-stimulating factor (GM-CSF), termed Talimogene laherparepvec (T-VEC; Imlygic™) [105]. Based on a randomized phase III clinical trial in which a significant improvement in durable and objective response rates were seen in patients with advanced melanoma, T-VEC became the first oncolytic virus to achieve regulatory approval in the United States, Europe and Australia [105, 106]. T-VEC replicates within neoplastic cells, and accumulation of the virions leads to lysis of the cancer cell, causing necrosis and cell death, releasing tumor-associated antigens and anti-tumor T cell responses, the local release of GM-CSF recruits dendritic cells and macrophages into the tumor and promotes their maturation allowing the presentation of tumor antigen to T cells in the regional lymph nodes, where stimulation of tumor-specific CD8+ T cells occurs, additional particles are released when tumor cells lyse, such as damage-associated molecular patterns and pathogen associated molecular patterns that also attract and stimulate inflammatory cells [105, 107, 108].

#### Cytokines

Cytokines, such as interferons, interleukins, chemokines, and growth factors, are immune modulators that are produced naturally by numerous cell types [109]. Certain cytokines can directly enhance or suppress T cell response against cancer cells, so it is not surprising that the systemic administration of cytokines (initially interferons and interleukins) was among the first approaches to cancer immunotherapy [110]. Early cytokine-based treatments were made possible by the development of recombinant DNA technology using genetically engineered Escherichia coli strains. This enabled the large-scale production of purified recombinant human cytokines that are suitable for systemic administration to patients.

Although IFN-α and IL-2 have been best characterized and used for cancer treatment, many additional cytokines are being investigated for use in cancer immunotherapy [110]; the discovery and early clinical use that interferon-α (IFN-α) was approved as therapy for hairy cell leukaemia and in 1995 it became the first immunotherapy approved by the US Food and Drug Administration (FDA) for the adjuvant treatment of stage IIB/III melanoma [87].

IL-2 is one of the key cytokines with pleiotropic effects on the immune system and it was an early candidate for cancer immunotherapy, approved for the treatment of metastatic renal cell carcinoma (1992) and later for metastatic melanoma (1998) by FDA. Although high doses of IL-2 showed promising results in metastatic renal cell carcinoma and melanoma, the toxicity and cost limited its application in a large population [110]. Thus, some investigators evaluated the efficacy of regimens containing low-dose IL-2 combined with other cytokines, such as interferon α (IFN-α).

Interferons are agents with antiviral, antiproliferative, and immunomodulatory properties. IFN-α has shown antitumor and antiviral efficacy and FDA approval was granted for the treatment of patients with hairy cell leukaemia, acquired immune deficiency syndrome-related Kaposi's sarcoma, and condylomata acuminata. Although IFNs are effective as single agents in certain clinical pathologic entities, increasing experience with these cytokines suggests that their greatest therapeutic potential may be realized in combination with other biological response modifiers, cytotoxic, or antiviral agents [110]. While IFN-α appears to be moderately effective in certain diseases, the flu-like syndrome associated with its use is a major limiting factor for its clinical application. It is notable to mention that the overwhelming majority of these interventions rely on T cells against tumors.

#### Cancer vaccines

Therapeutic vaccines represent a viable option for active immunotherapy of cancers that aim to treat late stage disease by using a patient's own immune system. The promising results from clinical trials recently led to the approval by the FDA of sipuleucel-T, a dendritic cell vaccine, for the treatment of stage IV metastatic but asymptomatic castrate-resistant prostate, the first therapeutic cancer vaccine [111]. Based on their format/content, they may be classified into several major categories, which include cell vaccines (tumor or immune cell), protein/peptide vaccines, and genetic (DNA, RNA and viral) vaccines [112].

One goal of cancer vaccines is to stimulate the immune system to attack and eradicate cancer cells. To this end, cancer vaccines contain whole cancer cells, parts of cancer cells, or purified antigens that enhance the immune response against cancer cells. In this context, cancer vaccines exhibit high specificity and low toxicity, but their therapeutic efficacy had been very low with a reported overall objective response rate of only 3.3%; tumor eradication has been achieved in models of cancer by intratumoral or peritumoral application of cytokines or by implantation of tumor cells expressing cytokines [113].

Autologous tumor vaccines prepared using patient-derived tumor cells represent one of the first types of cancer vaccines to be tested [114]. These tumor cells are typically irradiated, combined with an immunostimulatory adjuvant (e.g., BCG), and then administered to the individual from whom the tumor cells were isolated; one major advantage of whole tumor cell vaccines is its potential to present the entire spectrum of tumor-associated antigens to the patient's immune system [114]. However, preparation of autologous tumor cell vaccines requires sufficient tumor specimen, which limits this technology to only certain tumor types or stages [112, 114].

Allogeneic whole tumor cell vaccines typically contain two or three established human tumor cell lines, may be used to overcome many limitations of autologous tumor cell vaccines [115]. These include limitless sources of tumor antigens, standardized and large-scale vaccine production, reliable analysis of clinical outcomes, easy manipulation for expression of immunostimulatory molecules and cost-effectiveness [112]. However, two multi-institutional randomized phase III trials in patients with stage III and IV melanoma failed to achieve a determination of vaccine efficacy, and therefore, these trials were discontinued [116]. Tumor-infiltrating professional APCs are infrequent within the TME and these cells often show a tolerogenic phenotype with only low-level expression of co-stimulatory membrane proteins such as CD80 and CD86, which hinders efficient activation of antitumor T cells. It is likely that re-educating APCs to become mature APCs, as well as the development of new approaches to boost the recruitment and the activation of professional APCs, will improve the generation and the function of antitumor T cells [89]; for this reason, DCs have been used in the past by exposing these cells to some form of tumor antigen in vitro, and then returning antigen-loaded DCs to the patient to stimulate anti-tumor immunity [117–119]. Clinical trials of DC immunotherapy have suggested that this approach can result in significant stimulation of the immune response against many different forms of cancer [120–122] (Table 1).

**Table 1:** Examples of clinical trials testing vaccination with ex vivo DCs

Vaccine and antigen	Indication	Key observations
GM-CSF–IL-4 DCs with or without HLA-A^*^0201-restricted peptides or peptides alone	Metastatic prostate cancer	One of the first studies that tested the immunogenicity of DCs
GM-CSF–IL-4 DCs with peptides, tumour lysates or autologous tumour-eluted peptides	Stage IV melanoma, renal cell carcinoma and malignant glioma	Loading DCs with complex antigen preparations; Objective clinical responses
Blood DCs and idiotype antigens	Multiple myeloma	Immunogenicity of DCs; Tumour regression
Mature GM-CSF–IL-4 DCs and peptides	Stage IV melanoma	Well-controlled and validated vaccine manufacture process; Testing mature DCs; Immunogenicity; Objective clinical responses
CD34^+^ HPC-derived DCs and peptides	Stage IV melanoma	One of the first studies to test CD34^+^ HPC-derived DCs; Loading vaccines with a mixture of well-defined peptides; Durable immune responses in long-term survivors; Objective clinical responses
FLT3 ligand-expanded blood DCs and altered peptides	Advanced CEA^+^ cancer	Immunogenicity; Objective clinical responses
Immature GM-CSF–IL-4 DCs	Healthy volunteers	Antigen-specific inhibition of effector T cell function after injection of immature DCs
GM-CSF–IL-4 DCs and tumour lysates	Refractory pediatric solid tumors	Immunogenicity; Objective clinical responses
Mature cryopreserved GM-CSF–IL-4 DCs	Stage IV melanoma	Immunogenicity
DCs loaded with autologous tumour RNA	Colon cancer	Feasibility; Immunogenicity
DCs loaded with killed allogeneic tumour cells	Stage IV melanoma	Immunogenicity; Durable objective clinical responses; Long-term survival
Monocyte-derived DCs loaded with the NK T cell ligand α-galactosylceramide	Advanced cancer	Adjuvant effect of NK cell activation on CD8^+^ T cell-mediated immune response
Monocyte-derived DCs	Melanoma	*In vivo* identification of antigen-specific immune response by PET imaging in patients
Comparative study of CD34^+^ HPC-derived Langerhans cells versus monocyte-derived DCs	Melanoma	Langerhans cell-based vaccines stimulated significantly greater tyrosinase-HLA-A^*^0201 tetramer reactivity than the monocyte-derived DC vaccines
Type 1-polarized monocyte-derived DCs	Glioma	Combination of DC vaccination with polyICLC to trigger systemic inflammation driven by type I interferon family members

*CEA* carcinoembryonic antigen; *DC* dendritic cell; *IL-4* interleukin-4; *GM-CSF* granulocyte–macrophage colony-stimulating factor; *HLA* human leukocyte antigen; HPC haematopoietic progenitor cell; *NK cell* natural killer cell; *PET* positron emission tomography; *polyICLC* polyinosinic–polycytidylic acid stabilized with poly-L-lysine and carboxymethylcellulose

The availability of patient's samples or specimens and the complex procedure of preparing individualized vaccines greatly limit the broad use of autologous cancer vaccines, including whole tumor cells or DCs [112]. Recombinant vaccines, which are based on peptides from defined tumor-associated antigens, and usually administered together with an adjuvant or an immune modulator, clearly have advantages. MAGE-1 is the first gene that was reported to encode a human tumor antigen recognized by T cells [123]. Most peptide-based vaccines in clinical trials target cancer-testis antigens, differentiation-associated antigens, or certain oncofoetal antigens (CEA, MUC-1) [112]. Although these vaccines were able to induce antigen-specific T cell responses, clinical outcomes have been disappointing; for example, in the phase III study that led to the approval of ipilimumab, no difference in overall survival was observed in patients with unresectable stage III or IV melanoma between the ipilimumab group and ipilimumab plus gp100 group [124]. However, Schwartzentruber et, al. in 2011, reported encouraging results from a randomized phase III trial involving patients with stage IV or locally advanced stage III cutaneous melanoma) in which the group treated with the gp100 (210M) peptide in Montanide ISA-51 adjuvant plus IL-2 demonstrated a statistically significant improvement in overall clinical response (16% vs. 6%, *P* = 0.03), longer progression-free survival (2.2 months vs. 1.6 months, *P* = 0.008) and improved median overall survival (OS = 17.8 vs. 11.1 months; *P* = 0.06) compared with the IL-2 group [125].

### Drugs inducing metabolic changes in the tumor microenvironment

It is proposed that myeloid-derived suppressor cells (MDSCs) aberrantly infiltrate the TME and effectively promote T cell dysfunction through production of nitric oxide and reactive oxygen species and expression of indoleamine-2,3-dioxygenase (IDO) and arginase 1 in mice. In this context, IDO, a tryptophan-catabolizing enzyme plays a key role in the normal regulation of peripheral immune tolerance.

This was first suggested when inhibition of IDO in pregnant mice caused spontaneous immune rejection of allogeneic foetuses [126]. In tumors, inhibition of the IDO pathway is theorized to help ameliorate a state of immune privilege created by tumor cells enhancing endogenous T cell mediated response against the tumor [127, 128]. The mechanism of “cancer immunoediting” is the direct consequence of a T cell-dependent immunoselection process that drives the formation of IDO1+ tumors [129]. IDO1 inhibitors could be administered as co-therapeutic agents in the presence of redox regulators, IFN-γ, or anti-IL-6. Combining IDO1 drugs with the inhibition of specific transcription factors regulating IDO1 activity (e.g., AhR) may also improve the effectiveness and specificity of chemotherapies. Current genome editing and exome sequencing technologies offer promising new strategies to identify novel tumor-specific mutational antigens and thus expand the repertoire of tumor-specific immunotherapies [129].

### Cellular therapy of cancer

Recently, the chimeric antigen receptor T (CAR-T) has been identified as a potential target in several malignancies. CAR-T cells recognize specific tumor antigens in a MHC-independent manner, which lead to the activation and execution of its antitumor function [130]. Once CAR specifically binds with tumor-associated antigens, T cells are activated through the phosphorylation of immune receptor tyrosine-based activation motifs and subsequently induce cytokine secretion, T cell proliferation, and cytotoxicity [131]. Chimeric immunoreceptor-activated T lymphocytes perform cytotoxicity through two predominant pathways: (1) secretion of perforin and granzyme granules and (2) activation of death receptor signalling via Fas/Fas-ligand or TNF/TNF-R [131]. Many strategies have been employed to potentiate the functions of CAR-T cells. It has been demonstrated that CAR-T cells with multiple signalling receptors could improve amplification, cytokine production, and cytotoxicity of T cells, as well as reduce antigen-induced cell death in vitro and in vivo [132]. Based on this mechanism, CAR-T antigens in solid tumors, focusing on the common targets of EGFR, HER2, and mesothelin have been implemented in preclincal trials [130, 133]. Although the curative effect in CAR-T treatments of hematological malignancies are reported, the results of pilot clinical trials on solid cancers are below expectation. Several obstacles remain to be overcome for a successful application of CAR-T cells in solid tumor, including the lack of ideal TAAs, inefficient trafficking of CAR-T cells to tumor sites, hostile solid tumor microenvironment, and the risk of developing on-target/off-tumor toxicities [130, 133].

Adoptive cell therapy is a particularly promising approach that utilizes endogenous tumor-infiltrating lymphocytes (TIL), which are expanded in vitro from a surgically resected tumor and then re-infused back into the patient [134]. This therapy for metastatic melanoma patients is associated with a 20 % complete response lasting beyond 3 years [135, 136]. When adoptive TIL therapy was applied to other solid tumors, including those of the uterus, cervix, lung, and gastrointestinal tract, some patients also showed excellent clinical responses [136, 137].

One of the major constraints of TIL therapy is the complex TIL-manufacturing process. The procedure starts with multi-well cultures of tumor fragments or single-cell suspensions obtained from disaggregated tumors, in the presence of high dose of IL-2 [138]. After this initial culture lasting 3–5 weeks, the tumor reactivity of different wells is tested by coculturing TIL samples with autologous tumor cells, the reactive sublines are then chosen for large-scale secondary polyclonal expansion during two additional weeks to generate the final product, this method is known as the “selected TIL” approach and has been the basis of most of the TIL clinical trials performed in melanoma patients at the National Cancer Institute [138, 139].

TIL therapy will not most likely be a standalone therapy but will need to be part of a larger combination regimen with checkpoint inhibitors. The need to perform this combination may be also critical when using PD-1-selected TILs, given the fact that these cells maintain a relatively high expression of PD-1 after expansion [138].

### Other new immunotherapy drugs and unmet requirements

The use of combination therapies that integrate immunotherapy with chemotherapy, radiation therapy, and targeted molecular therapy are under active investigation. For example, pembrolizumab in combination with platinum-doublet chemotherapy was evaluated in KN-021, a multi-center phase I/II study, that demonstrated that the combination group statistically significant improved objective response rate of 55% compared with 29% for chemotherapy alone (P = 0.0016) in non-small cell lung carcinoma. The rate of objective responses was similar among patients with a PD-L1 TPS <1% (57%) and those with a score of 1% or greater (54%) [139]. The potential mechanism of action of this synergism may rely in two major ways: (a) inducing immunogenic cell death as part of its intended therapeutic effect; and (b) disrupting strategies that tumors use to evade the immune response. It is known that anthracyclines activate expression of the pattern recognition receptor toll-like receptor-3, the rapid secretion of type I IFNs, and the release of the chemokine CXCL10; a type I IFN gene signature predicted response to anthracycline therapy in breast cancer patients [140]. Loss of function polymorphisms in TLR4 or P2RX7 fail to impact clinical outcome in patients with non-small cell lung cancer, suggesting that tumor biology, chemotherapeutic agent, or both may influence whether tumor cell death is immunogenic, and which cell death pathway is activated. Similar results have been reported with nivolumab, atezolizumab and durvalumab; given these promising results, ongoing phase III studies are being conducted to evaluate first-line immunotherapy in combination with chemotherapy versus chemotherapy or immunotherapy in advanced NSCLS [141].

Despite these advances, obstacles still exist for the field of cancer immunotherapy; these include the inability to predict treatment efficacy and patient response; the need for additional biomarkers; the development of resistance to cancer immunotherapies; the lack of clinical study designs that are optimized to determine efficacy; and high treatment costs [142]. The field of cancer immunotherapy is expected to advance rapidly in the coming years, moving away from cancer immunotherapies that broadly activate the immune system toward more targeted approaches that enhance efficacy and reduce toxicity [133].

Since the responses are quite variable and anatomic imaging showing an increased tumor size (pseudoprogression) may occur, there is an urgent need to develop technologies and imaging approaches, which may implement immune response criteria. This may help clinicians to decide whether to continue, pause or interrupt the treatment.

### Targets and radiopharmaceuticals for imaging tumor-infiltrating cells

Imaging of the immune cells in tumor microenvironment is very challenging because many cell subtypes can coexist in different phases of activation, also playing different roles. Therefore, achievement of an accurate evaluation of TME and its cellular components is a very complex task. In vivo imaging currently offers quantitative and sensitive modalities that exploit long-lived tracers for metabolic phenotypes, specific targets relevant for therapy or critical for their effector function. In this paragraph we will highlight these aspects of imaging specific immune cell populations in cancer lesions.

A diverse range of molecular imaging techniques and cell-labelling strategies are available for preclinical and clinical studies. Modalities that are currently used in clinical settings include positron emission tomography (PET) and single-photon emission computed tomography (SPECT) radionuclide imaging, as well as non-nuclear imaging techniques e.g. magnetic resonance imaging, ultrasound. In preclinical settings, optical imaging techniques, e.g. fluorescence and bioluminescence play an important role, as well as photoacoustic imaging [143]. However, the penetration depth of the signals derived from these techniques is currently too low for detection of labelled immune cells in clinical practice, therefore the following paragraph will mainly focus on nuclear medicine imaging.

### Cell labelling strategies

In vivo tracking of a particular cell subset can be accomplished either by direct or indirect labelling. With the direct labelling approach is possible to isolate the cells and radiolabel them in vitro prior to re-administering them in the subject (ex vivo labelling) or to inject in vivo a radiopharmaceutical that binds to a membrane specific antigen (in vivo labelling). The indirect labelling method relies on the transduction of a reporter gene into the cells prior their reinfusion. This leads to the expression of a specific enzyme or transporter that can be exploited to image cells after administration of appropriate substrates or probes [144]. The use of such radioactive compounds, able to diffuse through the plasma membrane, is one of the most common direct strategies, especially in a clinical setting. However, also other imaging techniques are emerging as valid alternatives, but with limited success [145].

#### Ex vivo labelling

Direct cell/ex vivo labelling is routinely performed to radiolabel leukocytes for white blood cell scintigraphy. Cells are isolated from the blood of patients and incubated with either ^99m^Tc-hexamethylpropyleneamine oxime (^99m^Tc-HMPAO) or ^111^In-oxine prior to re-infusion [146]. This is a well-established technique and offers the advantage of a lower background, since the radiopharmaceutical is already inside the cells and the signal from its physiological uptake in non-target organs is significantly reduced. However, specific training and equipment is required and when trying to radiolabel specific immune cell subtypes, additional purification steps lead to a cumbersome and time-consuming procedure. Moreover, administered activity results to be low because of the small percentage of each cell subpopulation in the total white blood cells (WBCs) and because of leakage of the radiopharmaceutical as cells die, with the subsequent uptake in non-target tissues at later time point. Similar issues, like the dilution effect caused by cell division, have been also observed when trying to label cells using a non-radioactive probe, thus limiting the sensitivity of these approaches [147].

#### In vivo labelling

A much more specific and straightforward approach is to inject in the subject a radiopharmaceutical that is able to bind to specific antigens expressed on the plasma membrane of each immune cell subtype. In general, this is accomplished by using radiolabelled mAbs and it is a common trend to select a therapeutic one (e.g. PD-1/PD-L1) so that the immunotherapeutic drug and the radiopharmaceutical share the same target. This strategy has been explored also for other pathologies with promising results. However, non-specific uptake by non-target organs like liver, spleen and bone marrow is usually pronounced and together with the long plasma half-life of mAbs limit their use for early time points and with the most common short-lived radioisotopes. This leads to higher-radiation doses to patients and, in some cases, a suboptimal target-to-background ratio [148].

### Imaging tumor-infiltrating lymphocytes

#### Ex vivo labelling

Accumulation of lymphocytes in tumor lesions has been already shown after labelling with ^111^In-oxine, but those old study had no real follow-up mainly because of low sensitivity and poor spatial resolution of indium-111. To overcome these limitations radiolabelling with PET isotopes has been explored for image quality and quantitative imaging. First attempts with [^18^F] Fluorodeoxyglucose ([^18^F]FDG) trying to exploit glucose transporters were not successful because of slow accumulation of cells in the tumors, leakage of the radiopharmaceutical and high accumulation of injected cells in the lungs at early time points [149]. Zirconium-89 can be a suitable alternative, with its longer half-life (3.3 d) and can be used to radiolabel oxine or other compounds able to diffuse through the plasma membrane. Despite a low labelling efficiency, Sato et al. reported that ^89^Zr-oxine labelling of cytotoxic lymphocytes is feasible, but when compared with ^111^In-oxine it suffers from similar limitations. Indeed, the radioisotope is eventually released from cells causing accumulation in the bones with consequent bone marrow irradiation [150–153]. In a melanoma model, it was observed accumulation of cytotoxic cells in the tumor lesion, with reduction of tumor volume over time, nevertheless images are not very impressive, maybe due to the small number of cells infiltrating the tumor. Copper-64 is another valid alternative, due to its intermediate half-life (12.7 h) that has already been proposed to radiolabel WBCs in place of technetium-99m or indium-111 for PET applications [154]. This isotope can be delivered inside the cells through the use of pyruvaldehyde-bis(N4-methylthiosemicarbazone a lipophilic compound in a manner similar to HMPAO or oxine. Release of the radioactive compounds from the cytoplasm was observed also in this case, thus confirming that the ex vivo approach is still characterized by important limitations. Attempts to use ^64^Cu-gold nanoparticles previously trapped in the cytoplasm of T lymphocytes did not solve this issue, which currently is an open challenge.

Imaging of T cell trafficking can be also achieved using other modalities like magnetic resonance imaging. To this purpose, the most common approach is to use small iron oxide particles (SPIO) that have to be vehiculated inside the cells by electroporation, transfection agents or molecules able to penetrate the cell membrane [155]. Then, like other particles, they remain trapped in the cytoplasm. Studies performed with SPIO-labelled lymphocytes in mice bearing ovalbumin-expressing tumors demonstrated the feasibility of this approach. Cell viability was not significantly affected by the procedure and signal from ovalbumin-expressing tumors, due to lymphocyte infiltration, remained high up to 72 h [156]. However, limitation of SPIO-based techniques derives from possible alteration of biodistribution of labelled cells or from the dilution effect caused by cell division. This also applies to other particle or fluorine-19 based techniques, like ^19^F-perfluorcarbon. In these cases, the labeling compound enters the circulation and is generally metabolized by liver or RES thus providing altered images. For this reason, scan at late time points is not advisable [157].

#### In vivo labelling

Since the majority of ex vivo approaches suffers from low specificity and none or weak binding to a specific biomarker, in vivo methods proved to be the more promising even though more challenging. Indeed, in addition to the specific signal due to the presence of the target of interest, images will display also the unspecific signal that derives from physiologic biodistribution of the injected radiopharmaceutical. Still this approach is highly specific and easier to implement in clinical practice. If we focus on TILs, we know that after activation they express peculiar receptors that can be used as biomarkers to follow their trafficking. In particular, it is possible to produce different mAbs against their clusters of differentiation (CD antigens). This has already been performed, for example, to target CD3, CD4 or CD8, with both PET and SPECT radiopharmaceuticals. By following this very well established “magic bullets” concept it is possible to virtually target any receptor on the plasma membrane of TILs [158]. Another recent approach was described by Griessinger et al. that exploited the turnover of a ^64^Cu-mAb-TCR complex to stably radiolabel T cells and follow their homing in mice [159]. This approach is promising but still limited to preclinical studies. Finally, since many immunotherapeutics are mAbs-based, many attempts have been made to radiolabel those very same antibodies to develop radiopharmaceuticals that share the same target with the anti-cancer drug. This is a key example of how it could be possible to non-invasively evaluate the expression status of a specific biomarker and make the most appropriate therapeutic choice. This particularly important for mAbs against immune check-point inhibitors like anti-PD-1 or anti-PD-L1. These two antibodies have been radiolabelled with PET or SPECT isotopes with promising results, yet none of them was able to enter in the clinical practice [160, 161].

**Fig. 2:**
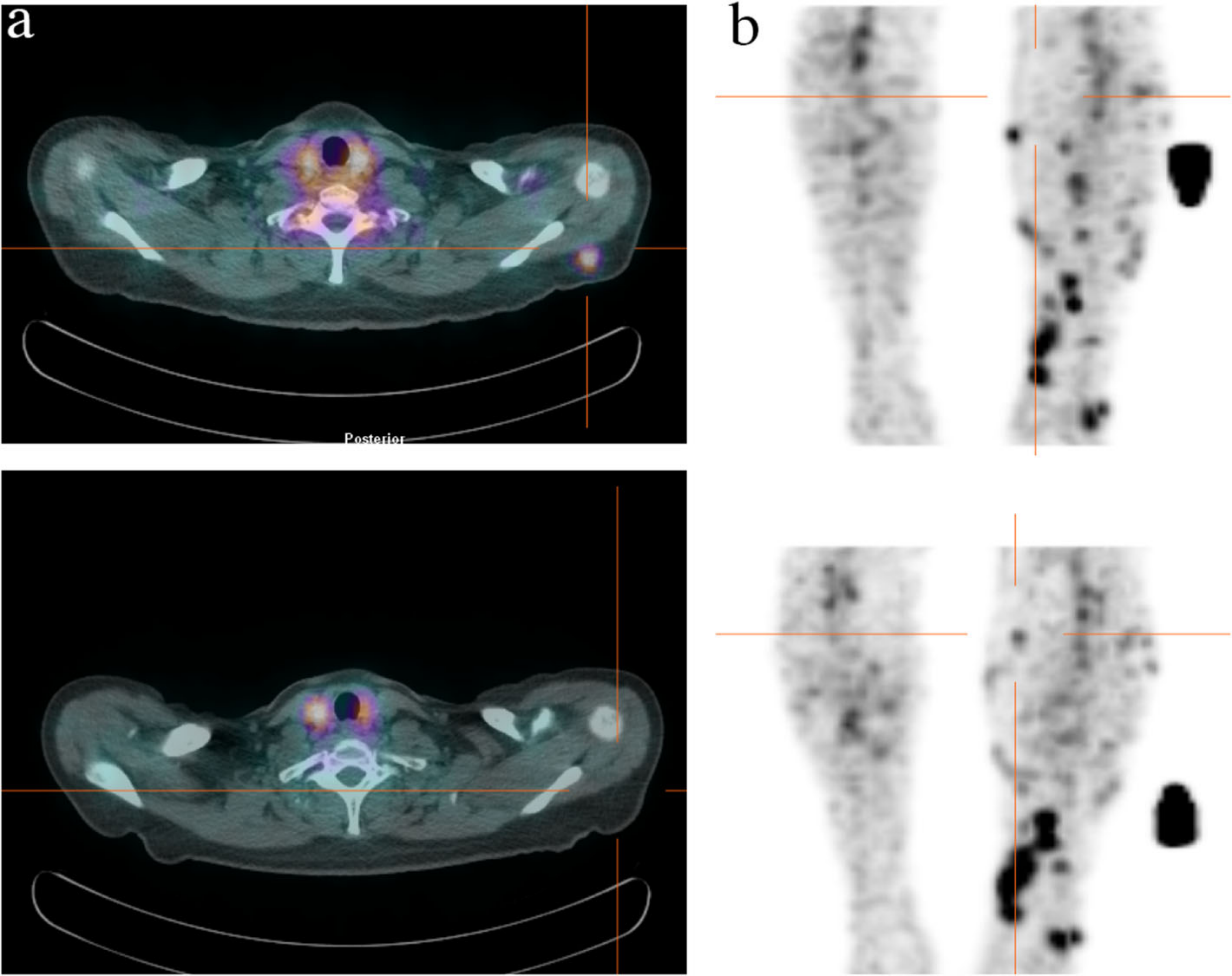
^99m^Tc-IL2 SPECT-CT in patients affected by metastatic melanoma before (top) and after (bottom) immunotherapy with ipilimumab. a) Patient with a ^99m^Tc-IL2-positive lesion that responded to therapy. b) Multimetastatic patient with different degree of uptake of ^99m^Tc-IL2

To overcome the long circulating half-life of mAbs, smaller molecules can be used and they include peptides or small proteins like cytokines. In particular, radiolabelled IL2 is one of the most studied cytokine-based radiopharmaceuticals. Its receptor, the CD25, is overexpressed on activated T lymphocytes and it drives their proliferation and inflammatory response. Therefore, radioactive IL-2 as a radiopharmaceutical to target T cells in vivo has been pioneered by Signore et al. in many autoimmune pathologies. A recent study, conducted in patients affected by metastatic melanoma and undergoing immunotherapy with either pembrolizumab or ipilimumab, demonstrated the feasibility of its use as a candidate-imaging tool to evaluate TILs into tumors [162]. Indeed, in some patients, lesions with high SUV at the pre-therapy scan positively responded to the therapy. However, what emerged from this study is that intra-patient heterogeneity is a true open challenge, since in the same patient, differential uptake in studied lesions over the course of the therapy were observed (Fig. 2). This leads to the need of more accurate studies in a higher cohort of patients, to understand common patterns of uptake and understand the mechanisms that cause therapy response or failure. As an alternative to intact mAbs, radiolabelled fragments like diabodies or minibodies offers a lower half-life (2-5 h or 5-12 h respectively) with faster clearance from the blood pool. However, lower specificity and stability is a common issue that should be taken into account.

This approach has been investigated by Tavarè et al. that developed an anti-CD8 cys-diabody radiolabelled with zirconium-89. This radiopharmaceutical showed specificity to activated T cells and allowed the authors to follow their infiltration of EL4-Ova tumors in an OT-I adoptive T cell therapy model. Moreover, they were able to demonstrate its potential by treating the same mice with an immune activating mAb (anti-CD137). Indeed, treated mice showed higher uptake of the radiopharmaceutical than controls, due to higher infiltration of tumor lesions [163, 164].

### Imaging tumor-infiltrating NK cells

#### Ex vivo labelling

Approaches to radiolabel tumor-infiltrating NK cells are similar to those described for T lymphocytes. Indeed, ^111^In-oxine, ^99m^Tc-HMPAO or [^18^F]FDG has been attempted to follow NK infiltration in patients undergoing immunotherapy or in pre-clinical models, but with limited success. Issues related to these techniques like poor sensitivity or altered biodistribution are amplified by the low number of NK cells and the cumbersome purification procedure prior their labelling and injection. This has been confirmed by Meller et al. that analysed NK cell number after 3 d from their administration in patients with renal cell carcinoma that received ^111^In-oxine-labelled and unlabelled NK cells from allogeneic donors [165]. They observed accumulation of labelled cells in two out of four metastases, but also significant circulating activity due to indium-111 released from dying cells. Other techniques like ^11^C-methyl-iodide or fluorescent labelling are described in the literature and potentially applicable, but they are still limited to early pre-clinical phases [166, 167]. Also, the use of SPIOs showed the typical signal reduction caused by cell division and decreased cell viability. From these studies emerged that injection of engineered NK cells against cancer specific antigens, was followed by a decrease in the signal at tumor site, thus confirming the strong anti-cancer activity of NKs and the potential of immunotherapies.

#### In vivo labelling

Very few papers describe the use of radiopharmaceuticals that binds to NKs in vivo. The most recent study investigated the use of ^99m^Tc-anti-CD56 mAb, being this antigen a distinctive marker of NK lineage. In the study, SCID mice bearing a human tumor xenograft derived from an aggressive cell line have been injected with NK cells, followed by injection of the radiopharmaceutical. Results showed uptake of the radiolabelled mAb in tumor lesions of mice that received NKs but not in controls that did not receive the cells. Immunohistochemistry confirmed the presence of tumor-infiltrating NK cells and their amount positively correlated with T/B ratios against the contralateral leg. In line with findings from other studies, the more the tumors were infiltrated, the more necrosis occurred due to active killing of cancer cells from NKs [168].

### Imaging tumor-associated macrophages

#### Ex vivo labelling

As for T and NK cells, macrophages can be cultured and differentiated ex vivo prior to radiolabelling with ^111^In-oxine or ^18^[F]FDG. However, in a study by Quillien et al., ^111^In-oxine-labelled macrophages, after in vitro expansion, accumulated in only 1 lesion out of 15 patients studied by SPECT imaging. They analysed cell’s phenotype after culturing them ex vivo and hypothesized that culturing conditions might have influenced their homing properties [169].

Given the innate phagocytic activity of macrophages, new approaches consist in the use of nanoparticles loaded with different reporter agents. For this purpose, SPIO nanoparticles were the most used for magnetic resonance imaging [170], but also ^19^F-loaded and/or fluorescent polymeric nanoparticles were used [171, 172]. All the limitations described above apply also for macrophage imaging, but the use of long-lived radioisotopes like zirconium-89 could lead to improved sensitivity and high T/B ratio. In the literature we can find rHDL, polymeric or cross-linked dextran nanoparticles radiolabelled with zirconium-89 and with different sizes. All of them showed high tumor uptake, but no correlation with number of TAMs subpopulations reflecting a possible unspecific uptake caused more from the EPR effect than from phagocytic activity [173, 174].

#### In vivo labelling

A well-known radiopharmaceutical for in vivo imaging of macrophages is the [^11^C]-(R)PK11195 that binds the translocator protein (TSPO) expressed at high grade in the mitochondrial membrane of macrophages and microglial cells. This has been mainly studied to image neuroinflammation, but may have application in imaging tumor-associated macrophages (TAMs). In vivo studies in mice that were not able to correlate the radiopharmaceutical uptake with TSPO expression, revealed by immunohistochemistry [175] and data in humans is very limited. An alternative approach has been proposed by Movahedi et al that used a ^99m^Tc-radiolabeled nanobody against the mannose receptor, which is expressed by macrophages. They were able to demonstrate uptake of the radiopharmaceutical in mannose receptor-expressing tumors as compared with control mice bearing negative tumors [176]. Similar results were obtained using a [^18^F]-SFB-counterpart that showed higher sensitivity and better biodistribution. A more recent approach exploits the use of 3′-Aza-2′-[^18^F]-fluoro-folic acid, also known as [^18^F]-AzaFol, which was previously used to image macrophages in various diseases [177]. This radiopharmaceutical has more advantages than TSPO, but its use in tumor associated macrophages has not been investigated yet.

### Indirect labelling

The indirect labelling approach is based on the insertion of a gene encoding for specific receptors or enzymes that allows the labelled probe to enter the cell and being specifically trapped inside. This strategy greatly reduces background and can be controlled by placing the genes under control of specific promotors. Moreover, the dilution effect is not an issue, since the construct will be maintained after cell division. On the other hand, it is very difficult to apply this strategy in clinical practice due to the need of genetic modification and cell manipulation.

The HSV1-tk reporter gene is a common technique that exploit the specificity of this enzyme for 9-[4-[^18^F]3-(hydroxymethyl)butyl] guanine ([^18^F]FHBG), 2-deoxy-2-[18F]5-ethyl-1-D-arabinofuranosyluracil ([^18^F]FEAU) or 2-deoxy-2-[^18^F]5-iodo-1-D-arabino-furanosyluracil ([^18^F]FIAU). These compounds are taken up by nucleoside transporters and then are phosphorylated by the enzyme remaining trapped in the cytoplasm [178]. This allows following cell trafficking in vivo by PET without the limitations of the short half-life of fluorine-18. This very same strategy can be applied by transducing the sodium/iodine symporter gene and administering iodine-124 for PET or sodium pertechnetate-99m for gamma camera imaging. However, this approach requires specific training and equipment due to genetic cell manipulation and is less suitable for routine human applications.

## Conclusion

In the present review, we wanted to give an overview of the immune cells that are involved in tumor microenvironment infiltration to highlight why imaging of their trafficking is so crucial with so many new immunotherapies entering the clinical practice. The main issue when evaluating tumor response to cancer immunotherapy is the enlargement due to infiltrating immune cells that eventually leads to tumor shrinkage and death. The same enlargement occurs in case of tumor progression due to cancer cell growth and in both situations increased uptake of [^18^F]FDG is observed. This limits the use of current available criteria and new ones are under definition with limited success. That is why we need new non-invasive tools to rely on and molecular imaging offers the most suitable approach.

Unfortunately, to fully achieve this goal we still have to face many open challenges like the many immune cell subtypes, small number and dynamic behaviour. This implies that radiopharmaceuticals of choice should be highly specific for biomarkers expressed by different immune cells. Molecular imaging can guide basic research, drug development and clinical follow up. It can also help researchers to elucidate mechanisms of pathology, effect of new drugs and predict efficacy of immunotherapies. There is still a long way to go, but many tools, summarized in Table 2 and 3, are already available and under investigation with different pros and cons. Antibodies are still “the magic bullets”, but their long circulating half-life and need of humanization at high costs are still the limiting factors. To overcome these issues fragments can be developed with loss of specificity but increased T/B ratio at earlier time points. This permits to use short-lived PET isotopes like gallium-68 or fluorine-18 in place of zirconium-89, thus reducing radiation dose to patients.

**Table 2:** Immunotherapeutic drugs approved for human use

Drug	Target	Clinical use	Mechanism of action	Labelling agent
**Rituximab**	CD20	B-Cell non-Hodgkin lymphoma, Chronic lymphocytic leukemia.	Direct induction of apoptosis.	^99m^Tc
**Ipilimumab/** **Tremelimumab**	CTLA-4	Metastatic melanoma, renal cell carcinoma, hepatocellular carcinoma.	Inhibition of CTLA-4 signaling	^64^Cu-DOTA
**Pembrolizumab/** **Nivolumab**	PD-1	Melanoma, non-small-cell lung cancer, renal cell carcinoma, Hodgkin lymphoma, squamous cell carcinoma of the head and neck, gastric cancer, cervical cancer, urothelial carcinoma, colorectal cancer with microsatellite instability-high (MSI-H) or mismatch repair deficient (dMMR) metastatic colorectal cancer.	Inhibition of PD-1 (expressed in lymphocytes), induction of tumor-specific T cell CD8+ activation against cancer	^64^Cu-DOTA; ^89^Zr-DFO; ^111^In-DTPA
**Atezolizumab**	PD-L1	Urothelial cancer, non-small cell lung cancer, small cell lung cancer, triple negative breast cancer.	Inhibition of PD-L1 (expressed in tumor cells), induction of tumor-specific T cell CD8+ activation against cancer	^89^Zr-DFO; ^111^In-DTPA
**Durvalumab**	PD-L1	Urothelial carcinoma, non-small cell lung cancer.	Inhibition of PD-L1 (expressed in tumor cells), induction of tumor-specific T cell CD8+ activation against cancer	^89^Zr-DFO
**Avelumab**	PD-L1	Merkel -cell carcinoma, renal cell carcinoma, urothelial carcinoma.	Inhibition of PD-L1 (expressed in tumor cells), induction of tumor-specific T cell CD8+ activation against cancer	^89^Zr-DFO
**Interleukin-2**	IL2 receptors	Metastatic renal cell carcinoma and metastatic melanoma	T cell activation and expansion	^123^I; ^99m^Tc; ^18^F
**Interferon alfa-2B**	INF- α receptors	Hairy cell leukemia, Malignant melanoma, follicular lymphoma, AIDS related Kaposi Sarcoma.	Immunomodulating activities, including cytotoxicity of lymphocytes. Upregulation of Th1 T-helper cell subsets	^131^I

**Source.**https://www.fda.gov/

**Table 3:** Other potential radiopharmaceuticals to image tumor infiltrating immune cells

Compound	Labelling agent	Target/Mechanism	Application
**T lymphocytes**	^111^In-oxine	Tumor infiltration/Cytokine production	Evaluation of immunotherapy/adoptive cell transfer efficacy
	^89^Zr-oxine		
	[^18^F]FDG		
	^64^Cu-gold nanoparticles		
	SPIO		
	^19^F-Perfluorcarbon		
**mAb-TCR-complex**	^64^Cu	Tumor infiltration	T cell homing
**Interleukin-2**	^123^I	Interleukin-2 receptors on activated lymphocytes	Evaluation of immunotherapy/adoptive cell transfer efficacy
	^99m^Tc		
	^18^F		
**Anti-CD8 cys diabody**	^89^Zr	CD8 on activated T cells	Evaluation of immunotherapy efficacy
**NK cells**	^111^In-oxine	NK cell infiltration	Evaluation of adoptive cell transfer efficacy – NK cell homing
	^89^Zr-oxine		
	[^18^F]FDG		
	SPIO		
**Anti-CD56 mAb**	^99m^Tc	CD56 on NK cells	Evaluation of adoptive cell transfer efficacy – NK cell homing
**Macrophages**	^111^In-oxine	Tumor infiltration by macrophages	Pre-clinical evaluation of TAMs
	^89^Zr-Nanoparticles		
	[^18^F]FDG		
	^19^F-Nanoparticles		
	SPIO		
**(R)PK11195**	^11^C	translocator protein (TSPO) expressed by TAMs	Pre-clinical evaluation of TAMs
**Anti-Mannose receptor nanobody**	^99m^Tc	Mannose receptor on TAMs	Pre-clinical evaluation of TAMs
	^18^F		

Radiolabelled immune checkpoint inhibitor mAbs showed great results in vivo, but to date they are still limited to pre-clinical studies. Also, cytokines like IL2 showed great potential and pilot human studies have already been performed with interesting results. The next step, for each of these radiopharmaceuticals, would be to increase the number of enrolled patients and define patterns of uptake to define new criteria for therapy decision-making and follow-up. This is particularly important, since inter- and intra-patient tumor heterogeneity is a real concern that may even require a “lesionalised therapy”.

This will require multimodal approaches as interactions in tumor microenvironment and different mechanisms of evasion from immune response are too complex to be unfolded by a single imaging tool.

## Abbreviations

APC: antigen-presenting cells
BCG: Bacillus Calmette-Guérin
CAF: Cancer associated fibroblasts
CAR-T: Chimeric antigen receptor T
CSF: Colony-stimulating factor
CXCL12: C-X-C motif chemokine 12
CTLA-4: Cytotoxic T lymphocyte associated protein 4
DC: Dendritic cells
ECM: Extracellular matrix
EMT: Epithelial mesenchymal transition
FDA: Food and Drug Administration
[^18^F]FDG: [^18^F]Fluorodeoxyglucose
GM-CSF: Granulocyte-macrophage colony-stimulating factor
HMPAO: Hexamethylpropyleneamine oxime
ICB: Immune checkpoint blockades
IDO: Indoleamine-2,3-dioxygenase
IFN-α: Interferon α
MHC: Major histocompatibility complex
MDSCs: Myeloid-derived suppressor cells
NK: Natural killer
NSCLC: Non-small cell lung cancer
PDAC: pancreatic ductal adenocarcinoma
PD-L1: Programmed death ligand 1
PD-1: Programmed death receptor 1
PET: Positron emission tomography
SPECT: Single-photon emission computed tomography
SPIO: Small iron oxide particles
TAMs: Tumor-associated macrophages
TAN: Tumor-associated neutrophils
TGFβ: transforming growth factor-β
TILs: Tumor-infiltrating lymphocytes
TME: Tumor microenvironment
TSPO: Translocator protein
VEGF: Vascular endothelial growth factor
WBCs: white blood cells
